# Effect of acupuncture and its influence on cerebral activity in patients with persistent asthma: study protocol for a randomized controlled clinical trial

**DOI:** 10.1186/s13063-020-04319-w

**Published:** 2020-05-14

**Authors:** Siyi Yu, Xiaohui Dong, Ruirui Sun, Zhaoxuan He, Chuantao Zhang, Mei Chen, Xiaojuan Hong, Lei Lan, Fang Zeng

**Affiliations:** 1 Brain Research Center, Acupuncture and Tuina School/Third Teaching Hospital, Chengdu University of Traditional Chinese Medicine, 37 Shierqiao Road, Chengdu, 610075 Sichuan China; 2 Hospital of Chengdu University of Traditional Chinese Medicine, Chengdu, 610072 Sichuan China; 3 Fifth People’s Hospital of Chengdu, Chengdu, 611130 Sichuan China

**Keywords:** Acupuncture, Asthma, Meridian–viscera relationship, Functional magnetic resonance imaging, Central mechanism

## Abstract

**Background:**

Previous studies suggested that acupuncture was a promising adjunctive treatment for asthma. However, the underlying mechanism of acupuncture for asthma remains unclear. The aim of the present trial is to explore whether and how specific meridian acupuncture works in quality of life and symptomatic improvement by modulating brain function in patients with asthma.

**Methods/design:**

This is a randomized controlled functional brain imaging trial currently being conducted in Sichuan, China. In total, 48 patients with mild to moderate persistent asthma will be recruited randomly and allocated to either of two acupuncture groups: acupuncture at the lung meridian or acupuncture at the heart meridian. The treatment period will last 4 weeks. The Asthma Quality of Life Questionnaire is the primary outcome. The Asthma Control Test, peak expiratory flow rate, forced expiratory volume in 1 s, Montreal Cognitive Assessment, Zung Self-rating Anxiety Scale, and Zung Self-rating Depression Scale will also be used to assess the clinical efficacy of different interventions. Functional magnetic resonance imaging (fMRI) will be performed to detect cerebral activity changes in each group. The clinical data and fMRI data will be analyzed between groups, then, the Pearson correlation analysis will be used to assess the association between the changes of cerebral activity features and the improvement of clinical outcomes in each group.

**Discussion:**

The present study has been established on the basis of the “meridian–viscera relationship” theory of traditional Chinese medicine and the modern central mechanism of acupuncture. The results of this trial would be useful to identify the efficiency of the specific meridian acupuncture for asthma. The investigation of its central mechanism would further expand knowledge of acupuncture for asthma.

**Trial registration:**

Chinese Clinical Trial Registry, ChiCTR1900027478. Registered on 15 November 2019.

## Background

Asthma is a chronic respiratory disease that affects about 300 million people worldwide, and 4.3% of the global population in younger adults (aged 18-45 years). More than 40% of patients with asthma are thought to have persistent symptoms that need long-term controller therapy [1–3]. The goals of asthma control are to minimize both the risk of adverse asthma outcomes and the symptom burden [3]. Most patients with mild to moderate asthma can be well controlled by medications such as inhaled corticosteroids, either alone or in combination with a long-acting β_2_-agonist [3]. However, patients with mild to moderate asthma remain at risk of asthma exacerbations and manifest considerable heterogeneity in asthma symptom control and health-related quality of life [4]. Thus, seeking complementary and alternative interventions attract both practitioners and patients [3].

Accumulating evidence indicates that acupuncture treatment can both significantly improve asthma-related quality of life (QoL) and lung function and decrease medication dosages for patients with asthma [5–10]. A recent meta-analysis including nine randomized controlled trials (RCTs) with a total of 777 patients demonstrated that the symptom response rate of conventional treatments plus acupuncture for asthma is statistically higher than that of conventional treatments alone [11]. These studies suggest that acupuncture may be a promising complementary option for asthma. However, the underlying mechanism of acupuncture for asthma remains unclear, which has limited its application in asthma control.

Although asthma results from airway inflammation, using magnetic resonance imaging (MRI) technique, researchers found that asthma is also characterized by cerebral structural and functional changes in some specific areas/circuits [12–17]. For example, using diffusion tensor imaging (DTI), Gao et al. found that patients with asthma show an abnormal structure in multiple white matter networks, including the frontolimbic network, and a significant correlation was found between these abnormal structural findings and clinical symptoms [17]. By functional magnetic resonance imaging (fMRI), these investigators found that patients with asthma manifested increased activity in the anterior cingulate cortex and insula, and the functional alterations in these brain regions are associated with the degree of lung inflammation [14]. These findings remarkably enhance understanding of the central pathophysiological mechanisms of asthma and provide the possibility and necessity to explore the central mechanism of acupuncture for asthma control.

With the guidance of the theory of the meridian–viscera association of traditional Chinese acupuncture, asthma has been recognized as a pulmonary disease, and the lung meridian is the most frequently used meridian for successful treatment of asthma. Therefore, the aims of this study are as follows: (1) to assess the therapeutic effects of acupuncture for persist asthma by comparing acupuncture at a specific meridian (the lung meridian) and nonspecific meridian (e.g., the heart meridian), (2) to explore the potential central mechanism of acupuncture for asthma using fMRI technique, and (3) to investigate the possible correlation between brain activity changes elicited by different acupuncture interventions and symptom and QoL improvements.

## Methods/design

**Fig. 1:**
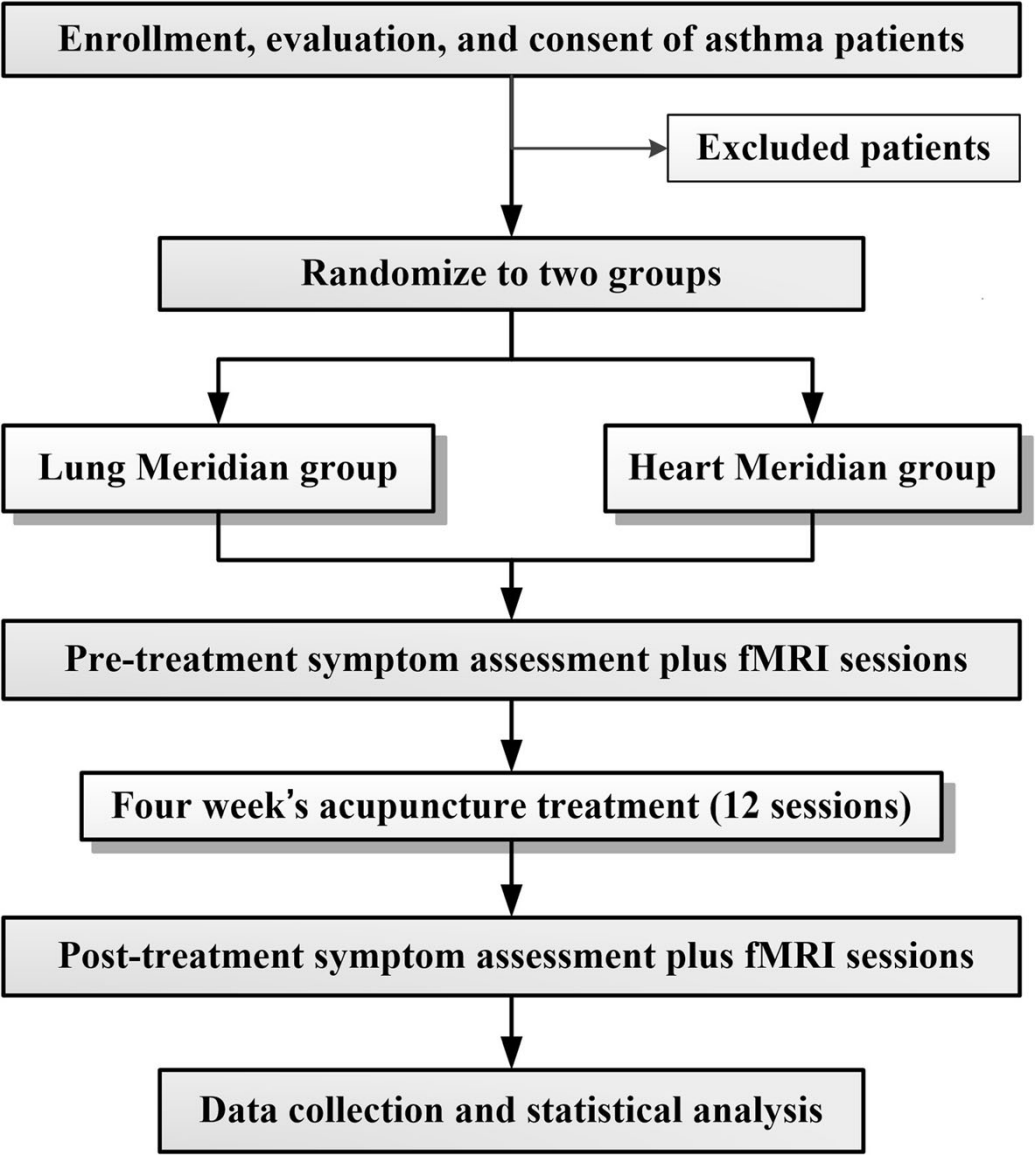
Flowchart of the trial. The present study is a randomized controlled neuroimaging trial. A total of 48 eligible patients will be randomized equally to either of two groups: the lung meridian group or the heart meridian group. During a 4-week treatment period, patients in the two acupuncture groups will receive 12 sessions of puncture treatments. Both the outcome assessments and functional magnetic resonance imaging (fMRI) scan will be performed at two time points: at baseline and at the end of the acupuncture treatments. The central mechanism of acupuncture in the treatment of asthma will be analyzed after data collection

This is a randomized controlled neuroimaging trial that will be performed at the First Affiliated Hospital of Chengdu University of Traditional Chinese Medicine (CDUTCM). A total of 48 patients with persistent asthma will be recruited and randomly allocated in a 1:1 ration to either of two acupuncture groups: group A (puncture at acupoints on the lung meridian) and group B (puncture at acupoints on the heart meridian). The treatment period will last 4 weeks. Clinical outcome measurements and fMRI scans will be assessed at baseline and at the end of treatment. The details of the study design are shown in Fig. 1.

The study protocol conforms to the Standard Protocol Items: Recommendations for Interventional Trials (SPIRIT) 2013 statement [18] and SPIRIT traditional Chinese medicine (TCM) 2018 extension statement [19]. The study has been approved by the First Affiliated Hospital of CDUTCM Institutional Review Board (approval no. 2019KL-045) and is registered with the Chinese Clinical Trial Registry (registration no. ChiCTR1900027478). All participants will provide voluntary written informed consent after a full discussion about the potential benefits and risks before participation. On the consent form, participants will be asked if they agree to use of their data, should they choose to withdraw from the trial. Participants will also be asked for permission for the research team to share relevant data with people from the universities taking part in the research or from regulatory authorities, when relevant. This trial does not involve collecting biological specimens for storage. YSY and XHD will recruit the patients and obtain informed consent before participation.

### Participants

#### Recruitment strategies

Patients with asthma will be recruited mainly from the outpatient clinic in the respiratory department of the First Affiliated Hospital of CDUTCM. Potential patients will also be recruited via advertisements, leaflets, and social media. All potential patients with asthma will undergo a physical examination and laboratory tests, such as lung function tests, x-rays, routine blood tests, and blood immune tests (immunoglobulin E). A respiratory physician will make the final diagnosis of potential patients. The diagnosis of asthma will be performed according to Western medicine definition, not using a specific TCM pattern.

#### Inclusion criteria

Eligible participants should fulfill all of the following criteria: (1) mild to moderate persistent asthma, according to the Chinese guideline for the prevention and management of bronchial asthma (version 2016) [20]; (2) aged from 18 to 65 years; (3) right-handed; (4) not participating in any other clinical trials in the past 1 month; and (5) signed informed consent.

#### Exclusion criteria

Patients matching any of the following criteria will be excluded: (1) diagnosed with other lung diseases, such as bronchiectasis, tuberculosis, lung abscess, cystic fibrosis, α_1_-antitrypsin deficiency, and restrictive lung disease, among others; (2) having aggravating malignant tumors or other clinically significant diseases that would jeopardize patient safety, such as uncontrolled heart failure, severe hypertension, or uncontrolled arrhythmias; (3) asthma occurring only when accidentally exposed to an allergen or chemical sensitizer; (4) being pregnant or lactating or those with childbearing requirements for nearly a half-year; (5) receiving acupuncture for asthma within the last 4 weeks; and (6) having MRI contraindications, such as having a heart pacemaker, having metallic foreign bodies, or having severe claustrophobia, among others.

### Sample size

The sample size calculation of the neuroimaging study is different from that of classic randomized controlled clinical trials. Power analyses for neuroimaging studies rely on assumptions about blood oxygenation level–dependent (BOLD) signal amplitude, smoothness, brain location, and other factors that render principled a priori designations difficult. It has been suggested that for fMRI studies, a minimum sample size (*n* = 20) should be used in order to obtain 80% power with an error threshold of 0.002 at a single-voxel level [21]. Considering a 20% dropout rate, the sample size in this study was increased to 24 per group, for a total of 48 participants needed.

### Randomization

After the baseline assessment, the eligible participants will be randomly allocated with an equal ratio to their respective groups. To avoid bias in researchers subjective factors, randomization will be implemented by a clinical information management system (Beijing Bioknow Information Science & Technology Co. Ltd., Beijing, China). When participant-recruiting staff members decide to recruit an eligible patient with asthma, they will send the patient’s name, sex, age, and telephone number to this system online. Then, the randomized result will be delivered to the acupuncturists.

### Blinding

Because of the different acupoints in the two groups, the acupuncturists will not be blinded to group allocation. Patients in the two groups will be separated into cubicles to refrain from communication. Outcome assessors and statistical analysts will be blind to the procedure and the results of randomization, group allocation, and intervention.

### Interventions

Subjects receive regular medical therapy (budesonide/formoterol [Symbicort; AstraZeneca, Cambridge, UK] or fluticasone/salmeterol [Seretide; GlaxoSmithKline, Brentford, UK]) as usual while participating in this study, except that changing the asthma control medication is not allowed during this study. On the basis of regular therapy, patients with asthma randomly allocated will receive two different acupuncture treatments: puncture at acupoints on the lung meridian or acupoints on the heart meridian.

#### Acupuncture intervention

According to records in the TCM ancient work *Huangdi Neijing*, acupuncture points belonging to the lung meridian have a noticeable effect on lung disease. Therefore, we chose the following acupuncture points: Patients in group A will receive manual acupuncture at six acupoints belonging to the lung meridian (bilateral *Taiyuan* [LU9], *Lieque* [LU7], *Chize* [LU5]) with disposable sterile filiform needles (0.25 × 25 mm, 0.35 × 40 mm; Huatuo Medical Instrument Co., Ltd., Suzhou City, China). Patients in group B are the reference group, and they will receive manual acupuncture at six acupoints belonging to the heart meridian (bilateral *Shenmen* [HT7], *Yinxi* [HT6], *Shaohai* [HT3]). The manipulations are as follows: Needles will be inserted into acupoints at a depth of 20–30 mm after skin disinfection using alcohol; acupuncturists will then bidirectionally twist needles by 90–180 degrees, lifting and thrusting needles with the amplitude of 3–5 mm for 1–1.5 Hz to induce *deqi* sensation. After the *deqi* sensation is attained, needles will be retained at the acupoints for 30 min. During the 30 min, the above procedures will be manipulated intermittently to maintain the *deqi* sensation.

Patients in both groups will receive a total of 12 sessions of acupuncture in 4 weeks with 3 sessions per week. All the acupuncture manipulation will be performed by two licensed acupuncturists with at least 3 years of clinical experience.

The acupuncture treatment will be conducted by doctors in the Department of Acupuncture and Tuina School of CDUTCM, with more than 6 years of TCM college education and at least 4 years of clinical experience.

#### Regular medical therapy

During the study period, all participants will receive regular medical therapies according to the Chinese guideline for the prevention and management of bronchial asthma. Asthma medications such as cromolyn sodium, nedocromil sodium, leukotriene modifiers, and theophylline require a stable dose for 3 months before enrollment. Nasal glucocorticoids and antihistamines require a stable dose for 2 months before enrollment. A short course of antihistamines or nasal corticosteroids is allowed to treat pollinosis. Desensitization treatment in the maintenance period requires a stable dose for at least 1 month before enrollment. During the study, short-term (< 10 days) use of systemic corticosteroids and temporary use of aerosolized therapy (including β_2_-agonists, anticholinergics, and steroids) and antibiotic therapy were allowed for acute asthma attacks. All medications used need to be maintained at a stable dose throughout the study period. Furthermore, a salbutamol metered-dose inhaler (100 mg per puff) will be provided as rescue medication throughout the trial.

### MRI data acquisition

MRI data will be acquired with a 3.0-T MRI scanner (Siemens AG, Erlangen, Germany) at Huaxi Magnetic Resonance Research Center, West China Hospital of Sichuan University, Chengdu, China. All patients will be asked to stay awake, keep their eyes open, and remain still during the fMRI can. The participant’s head should be placed in the head mask, and a sponge will be inserted to strengthen the fixation of the head. The scanning procedure contains a localizer, high-resolution three-dimensional T1-weighted imaging (3D-T1WI), BOLD-fMRI, and a DTI sequence. The 3D-T1WI scanning parameters will be as follows: repetition time (TR)/echo time (TE) = 1900/2.26 ms; slice thickness = 1 mm; slice number = 30; matrix size = 128 × 128; and field of view (FOV) = 256 × 256 mm. The BOLD-fMRI scanning parameters will be as follows: TR/TE = 2000/30 ms; flip angle = 90 degrees; slice number = 30; matrix size = 128 × 128; FOV = 240 × 240 mm; slice thickness = 5 mm; and total volume = 240. The DTI data will be acquired with the following parameters: FOV = 240 × 240 mm; TR/TE = 6800/93 ms; matrix size = 128 × 128; and slice thickness = 3 mm with no gap. Two diffusion-weighted sequences were acquired using gradient values b = 1000 s/mm^2^ and b = 0 with the diffusion-sensitizing gradients applied in 64 noncollinear directions. All images will be checked by a consultant radiologist at West China Hospital of Sichuan University to exclude unexpected brain lesions in recruits.

### Outcomes

The primary outcome measurements are the Asthma Quality of Life Questionnaire (AQLQ) [22] for assessing health-related impairment of QoL in adult patients with asthma [23]. AQLQ contains 32 items, including 4 domains: activity limitation, symptoms, mental health, and environmental stimuli. It will take 5–10 min for the assessor to complete the AQLQ with each participant.

The secondary outcome measurements include the Asthma Control Test (ACT), the peak expiratory flow rate (PEFR), forced expiratory volume in 1 s (FEV_1_), Montreal Cognitive Assessment (MoCA), Zung Self-rating Anxiety Scale (SAS), and Zung Self-rating Depression Scale (SDS). The ACT is a questionnaire consisting of five questions with a 5-point scale for each question, and it is validated for assessing asthma control [24]. PEFR and FEV_1_ are the main and objective monitors for asthma therapy. Furthermore, this trial also selects SAS, SDS, and MoCA as the secondary clinical outcomes to assess the emotional state and general cognitive function for psychological cognition importance in the pathogenesis of the patient’s asthma.

All outcomes will be assessed at baseline and at the end of treatment. An overview of the outcome measurement at different time points is shown in Table 1.

**Table 1:** Study schedule

	Baseline	Allocation	Treatment phase			
	Week 0	Week 2	Week 3	Week 4	Week 5	Week 6
Eligibility screen	√					
Inclusion/exclusion criteria	**√**					
Informed consent	**√**					
Demographics	**√**					
Medical history	**√**					
Physical examination	**√**					
Randomization		**√**				
**Interventions**						
Group A (acupuncture at lung meridian)			**√**	**√**	**√**	**√**
Group B (acupuncture at heart meridian)			**√**	**√**	**√**	**√**
**fMRI scan**						
Group A (acupuncture at lung meridian)		**√**				**√**
Group B (acupuncture at heart meridian)		**√**				**√**
**Assessments**						
AQLQ		**√**				**√**
ACT		**√**				**√**
PEF		**√**				**√**
SAS		**√**				**√**
SDS		**√**				**√**
MoCA		**√**				**√**
**Participants safety**						
Laboratory test		**√**				**√**
Adverse events			**√**	**√**	**√**	**√**

Abbreviations: *AQLQ* Asthma Quality of Life Questionnaire, *ACT* Asthma Control Test, *FEV*_*1*_ Forced expiratory volume in 1 s, *MoCA* Montreal Cognitive Assessment, *PEF* Peak expiratory flow rate, *SAS* Self-rating Anxiety Scale, *SDS* Self-rating Depression Scale

### Patient safety

Safety monitoring will be conducted throughout the trial with reporting of adverse events (AEs) and serious adverse events (SAEs) in each participant’s case report form (CRF). All AEs/SAEs will be reported immediately to the study principal investigator and attending physician. Each AE will be recorded separately.

### Data management

Clinical data will be managed with printed and electronic CRFs. Only outcome assessors have access to CRFs and will perform data double-entry. The researchers will be required to follow the requirements of the CRF and fill in the relevant information in a timely and accurate manner. The evidence-based medicine center of the CDUTCM will be responsible for monitoring the study and data every 3 months and will make the final decision to terminate the trial.

### Data analysis

The primary analysis will be conducted on a per-protocol basis. The data analysis will be completed by statisticians who are independent of the research and blind to the group assignments. For nonimaging data, statistical analyses will be conducted using IBM SPSS 23.0 Statistics software (IBM Corporation, Armonk, NY, USA). For clinical information, data will be presented as means with SDs. Student’s *t* test and the chi-square test will be used to compare group differences at baseline. A paired *t* test will be used to compare the clinical outcomes within the groups. Analysis of variance and the Kruskal-Wallis test will be used to compare the clinical feature changes between groups. The significance will be set at the 5% level with two-sided tests. The clinical and neuropsychological data, including AQLQ, ACT, MoCA, SAS, and SDS, will be converted to domain z scores for correlational analysis with imaging data.

The MRI data will be preprocessed and analyzed by statistical parametric mapping with SPM12 (http://www.fil.ion.ucl.ac.uk/spm/) and the CONN functional connectivity toolbox 18b (https://web.conn-toolbox.org/) using MATLAB 2014b software (MathWorks, Inc., Natick, MA, USA). The structural MRI data will be analyzed using the VBM toolbox within SPM12. The steps include checking for artifacts, structural abnormalities, and pathologies; image segmenting; normalizing to standard template; and spatial smoothing. The preprocessing steps of fMRI data include slice timing correction; head motion correction; skull stripping using the Brain Extraction Tool BET (https://fsl.fmrib.ox.ac.uk/fsl/fslwiki/BET); coregistration of the anatomical image to the mean functional image; segmentation of the anatomical gray matter, white matter, and cerebrospinal fluid; normalization to the MNI152 standard template; smoothing; and band-pass filtering. After preprocessing, a series of brain activity information, including amplitude of low-frequency fluctuation, group independent component analyses, seed-based functional connectivity, complex network analyses, and dynamic connectivity analyses, will be calculated to investigate the brain response to different treatment. Pearson correlation analysis will be used to assess the association between the changes of cerebral activity features and the improvement of clinical outcomes in each group. Finally, mediation analysis will be employed to detect whether the effect of acupuncture treatment on the symptoms and QoL is mediated through regulating the cerebral function. In the mediation analysis, the acupuncture method is the independent variate (X), the cerebral function is the mediator (M), and the symptoms and QoL alteration are the dependent variates (Y).

## Discussion

Established on the basis of the “meridian–viscera relationship” theory of TCM and modern central mechanism of acupuncture, the present randomized controlled neuroimaging study is the first to explore whether and how specific meridian acupuncture works in improving QoL and symptoms by modulating the altered brain function in patients with asthma.

### Study feasibility

In recent years, brain imaging techniques were applied to investigate the physiopathology of asthma and revealed structural and functional abnormalities in brain circuits involved in the pathogenesis of asthma [13–17, 25]. These studies provided an approach to investigate the potential mechanism of some interventions for asthma control. Since then, a few longitudinal studies have begun to uncover functional alterations in the brain due to treatment for asthma. For instance, Yu et al. [26] investigated the spontaneous brain activity with resting-state fMRI before and after cognitive behavioral therapy (CBT) in adult patients with asthma. They reported that the beneficial effects of CBT on asthma control were associated with the reversed abnormal spontaneous brain activity in the bilateral occipital lobe and sensorimotor cortex.

Central integration has been considered as an essential mechanism acupuncture’s therapeutic effect since the 1970s [27, 28]. In the past 20 years, with the aid of functional brain imaging techniques such as fMRI, positron emission tomography, electroencephalography, and magnetoencephalography [29], investigators have mapped the cerebral regions/circuits/networks participating in acupuncture effects for treating neuropathies [30–33], gastrointestinal disorders [34–36], and motor diseases [37, 38]. These studies provided abundant visual evidence for understanding the central mechanism of acupuncture.

Although acupuncture-related neuroimaging studies of patients with asthma have not yet been conducted, the proven central pathological features of asthma and the rich experience of acupuncture neuroimaging studies provide a basis for investigating the central mechanism of acupuncture in the treatment of asthma in this study.

This study is concerned with the relative specificity of the “meridian–viscera correlation” and is expected to provide evidence for the optimization of acupuncture treatment for asthma. In traditional meridian theories, “meridian–viscera correlation” is an essential principle for acupuncture in the treatment of viscera-related diseases [39]. It means that each meridian pertains to some specific viscera and connects with several viscera. When the viscera are affected, the acupoints on the related meridian can be selected for the affected viscera. For example, the lung meridian pertains to the lung, so the acupoints on the lung meridian can be used for lung diseases. The “meridian–viscera correlation” theory has also been proven by modern clinic trials. Our previous RCT demonstrated that, compared with the acupoint on the gallbladder meridian, acupuncture at the stomach meridian showed a better effect in improving QoL and relieving symptoms for patients with functional dyspepsia (FD) [40], indicating that the stomach meridian is the best choice for treating stomach disease. Our later neuroimaging studies [41] also demonstrated that puncturing at the acupoints on the stomach meridian elicited wider and more remarkable positive modulation in the abnormal cerebral function in patients with FD than that seen for acupoints on the gallbladder meridian. So, this study focuses on the difference in efficacy and cerebral responses resulting from different acupuncture interventions and tries to provide evidence for the “meridian–viscera correlation” theory.

### Quality control program

The quality control program is the precondition for the reliability of our results. Our previous study indicated that strict quality control plays an essential role in the guarantee of high repeatability in acupuncture neuroimaging studies [42]. Accordingly, we designed a quality control program focused on the homogeneity of participants, standardization of fMRI scans, and strictness of acupuncture procedures to improve the reproducibility and reliability of the results.

First, one of the important considerations in the neuroimaging study is that the neural activity in humans is highly variable. The connectivity topology of the brain can be influenced by potentially confounding factors such as age [43], sex [44], handedness [45], and even mental state [46]. Hence, rigorous inclusion/exclusion criteria at the stage of participant enrollment will be established in this study. For example, patients with asthma will be restricted to ages between 40 and 65 years and to being right-handed to guarantee the homogeneity of the clinical population. Concurrently, the SAS and SDS scores will be recorded to evaluate the patients’ psychological state and exclude patients with severe anxiety and depression.

The sensitivity to subject motion is another challenge in fMRI studies [47]. Even the slightest source of artifact, such as physiological fluctuations (respiration and cardiac fluctuations) as well as head motion, can highly influence the final estimate of connectivity. Hence, the patients will be asked to stay awake, keep their eyes open, and remain still at the stage of the fMRI scan. To eliminate possible head motion, the participant’s head should be placed in the head mask, and a sponge will be inserted to strengthen the fixation of the head.

Finally, the acupuncture manipulation will be performed by two experienced acupuncturists with a strict and standard operative procedure. The *deqi* sensation will be evaluated and recorded with a visual analogue scale after each acupuncture treatment.

The limitations of this study lie primarily in the seasonality of asthma attacks [48] and the diversity of routine medical treatment for patients with asthma [49]. Thus, inconsistency of regular treatments for asthma may confound our results.

In summary, this fMRI trial is designed to investigate the clinical and cerebral activity changes evoked by different meridians (the acupoints on the lung meridian vs. the acupoints on the heart meridian) in patients with mild to moderate persistent asthma and to explore the associations between the cerebral activity changes and clinical variable changes. The results of this trial will help to provide the optimal adjunctive therapeutic approach for improving the QoL of adults with persistent asthma and provide visual evidence for the clinical application of acupuncture for asthma management.

## Trial status

This trial was registered with the Chinese Clinical Trial Registry (www.chictr.org.cn) on 15 November 2019 (registration number ChiCTR1900027478; protocol version 2.0). The patient recruitment of this trial has begin on 1 December 2019, 16 patients with asthma have been enrolled up to now (12 May 2020).

## Abbreviations

ACT: Asthma Control Test
AE: Adverse event
AQLQ: Asthma Quality of Life Questionnaire
BOLD: Blood oxygenation level-dependent
CBT: Cognitive behavioral therapy
CDUTCM: Chengdu University of Traditional Chinese Medicine
CRF: Case report form
DTI: Diffusion tensor imaging
3D-T1WI: Three-dimensional T1-weighted imaging
FD: Functional dyspepsia
FEV_1_: Forced expiratory volume in 1 s
FOV: Field of view
MoCA: Montreal Cognitive Assessment
MRI: Magnetic resonance imaging
PEFR: Peak expiratory flow rate
QoL: Quality of life
RCT: Randomized controlled trial
SAE: Serious adverse event
SAS: Zung Self-rating Anxiety Scale
SDS: Zung Self-rating Depression Scale
TCM: Traditional Chinese medicine
TE: Echo time
TR: Repetition time

## References

[1] Masoli M, Fabian D, Holt S, Beasley R (2004). Global Initiative for Asthma (GINA) Program. The global burden of asthma: executive summary of the GINA Dissemination Committee report. Allergy..

[2] Heaney LG, Robinson DS (2005). Severe asthma treatment: need for characterising patients. Lancet.

[3] Papi A, Brightling C, Pedersen SE, Reddel HK (2018). Asthma. Lancet.

[4] Calhoun WJ, Haselkorn T, Mink DR, Miller DP, Dorenbaum A, Zeiger RS (2014). Clinical burden and predictors of asthma exacerbations in patients on guideline-based steps 4–6 asthma therapy in the TENOR cohort. J Allergy Clin Immunol Pract.

[5] Brinkhaus B, Roll S, Jena S, Icke K, Adam D, Binting S (2017). Acupuncture in patients with allergic asthma: a randomized pragmatic trial. J Altern Complement Med.

[6] Choi JY, Jung HJ, Kim JI, Lee MS, Kang KW, Roh YL (2010). A randomized pilot study of acupuncture as an adjunct therapy in adult asthmatic patients. J Asthma..

[7] Biernacki W, Peake MD (1998). Acupuncture in treatment of stable asthma. Respir Med.

[8] Reinhold T, Brinkhaus B, Willich SN, Witt C (2014). Acupuncture in patients suffering from allergic asthma: is it worth additional costs?. J Altern Complement Med.

[9] Chu KA, Wu YC, Ting YM, Wang HC, Lu JY (2007). Acupuncture therapy results in immediate bronchodilating effect in asthma patients. J Chin Med Assoc.

[10] Pai HJ, Azevedo RS, Braga AL, Martins LC, Saraiva-Romanholo BM, de Arruda Martins M (2015). A randomized, controlled, crossover study in patients with mild and moderate asthma undergoing treatment with traditional Chinese acupuncture. Clinics (Sao Paulo).

[11] Jiang C, Jiang L, Qin Q (2019). Conventional treatments plus acupuncture for asthma in adults and adolescent: a systematic review and meta-analysis. Evid Based Complement Alternat Med.

[12] Busse WW (2012). The brain and asthma: what are the linkages?. Chem Immunol Allergy.

[13] von Leupoldt A, Sommer T, Kegat S, Eippert F, Baumann H, Klose H (2009). Down-regulation of insular cortex responses to dyspnea and pain in asthma. Am J Respir Crit Care Med.

[14] Rosenkranz MA, Busse WW, Johnstone T, Swenson CA, Crisafi GM, Jackson MM (2005). Neural circuitry underlying the interaction between emotion and asthma symptom exacerbation. Proc Natl Acad Sci U S A..

[15] Bian R, Zhang Y, Yang Y (2018). White matter integrity disruptions correlate with cognitive impairments in asthma. J Magn Reson Imaging.

[16] Li QG, Zhou FQ, Huang X, Zhou X, Liu C, Zhang T (2018). Alterations of resting-state functional network centrality in patients with asthma: evidence from a voxel-wise degree centrality analysis. Neuroreport..

[17] Gao X, Xiao Y, Lv P, Zhang W, Gong Y, Wang T (2019). Altered brain network integrity in patients with asthma: a structural connectomic diffusion tensor imaging study. Respir Physiol Neurobiol.

[18] Chan AW, Tetzlaff JM, Altman DG, Laupacis A, Gøtzsche PC, Krleža-Jerić K (2013). SPIRIT 2013 statement: defining standard protocol items for clinical trials. Ann Intern Med.

[19] Dai L, Cheng CW, Tian R, Zhong LL, Li YP, Lyu AP (2019). Standard protocol items for clinical trials with traditional Chinese medicine 2018: recommendations, explanation and elaboration (SPIRIT-TCM Extension 2018). Chin J Integr Med..

[20] Chinese Thoracic Society Asthma Workgroup (2016). Chinese guideline for the prevention and management of bronchial asthma (version 2016). Chin J Tubercul Respir.

[21] Desmond JE, Glover GH (2002). Estimating sample size in functional MRI (fMRI) neuroimaging studies: statistical power analyses. J Neurosci Methods.

[22] Juniper EF, Guyatt G, Epstein R, Ferrie PJ, Jaeschke R, Hiller T (1992). Evaluation of impairment of health related quality of life in asthma: development of a questionnaire for use in clinical trials. Thorax..

[23] Testa MA, Simonson DC (1996). Assessment of quality-of-life outcomes. N Engl J Med.

[24] Nathan RA, Sorkness CA, Kosinski M, Schatz M, Li JT, Marcus P (2004). Development of the asthma control test: a survey for assessing asthma control. J Allergy Clin Immunol.

[25] Xiong X, Zhu H, Wang T, Ji Y (2016). Altered intrinsic regional brain activity in female asthmatics with or without depressive symptoms: a resting-state functional magnetic resonance imaging study. J Asthma.

[26] Yu SY, Lv ZT, Zhang Q (2017). Electroacupuncture is beneficial for primary dysmenorrhea: the evidence from meta-analysis of randomized controlled trials. Evid Based Complement Alternat Med.

[27] Han JS, Ho YS (2011). Global trends and performances of acupuncture research. Neurosci Biobehav Rev.

[28] Yang ES, Li PW, Nilius B, Li G (2011). Ancient Chinese medicine and mechanistic evidence of acupuncture physiology. Pflugers Archiv..

[29] He T, Zhu W, Du SQ (2015). Neural mechanisms of acupuncture as revealed by fMRI studies. Auton Neurosci.

[30] Napadow V, Makris N, Liu J, Kettner NW, Kwong KK, Hui KK (2005). Effects of electroacupuncture versus manual acupuncture on the human brain as measured by fMRI. Hum Brain Mapp.

[31] Duan G, He Q, Pang Y, Chen W, Liao H, Liu H, et al. Altered amygdala resting-state functional connectivity following acupuncture stimulation at BaiHui (GV20) in first-episode drug-naïve major depressive disorder. Brain Imaging Behav. 2019. 10.1007/s11682-019-00178-5. [Epub ahead of print]. 10.1007/s11682-019-00178-5 31432318

[32] Li Y, Wang Y, Liao C, Huang W, Wu P (2017). Longitudinal brain functional connectivity changes of the cortical motor-related network in subcortical stroke patients with acupuncture treatment. Neural Plast.

[33] Wu P, Zhou YM, Liao CX, Tang YZ, Li YX, Qiu LH (2018). Structural changes induced by acupuncture in the recovering brain after ischemic stroke. Evid Based Complement Alternat Med.

[34] Zeng F, Qin W, Ma T, Sun J, Tang Y, Yuan K (2012). Influence of acupuncture treatment on cerebral activity in functional dyspepsia patients and its relationship with efficacy. Am J Gastroenterol.

[35] Zhou S, Zeng F, Liu J, Zheng H, Huang W, Liu T (2013). Influence of acupuncture stimulation on cerebral network in functional diarrhea. Evid Based Complement Alternat Med.

[36] Liu CZ, Xie JP, Wang LP, Liu YQ, Song JS, Chen YY (2014). A randomized controlled trial of single point acupuncture in primary dysmenorrhea. Pain Medicine.

[37] Kong J, Wang Z, Leiser J, Minicucci D, Edwards R, Kirsch I (2018). Enhancing treatment of osteoarthritis knee pain by boosting expectancy: a functional neuroimaging study. Neuroimage Clin.

[38] Chen J, Wang Z, Tu Y, Liu X, Jorgenson K, Ye G (2018). Regional homogeneity and multivariate pattern analysis of cervical spondylosis neck pain and the modulation effect of treatment. Front Neurosci.

[39] Zhou Y, Wang K, Ling H, Zhou M, Wu Z, Cai R, Xia Y, Ding G, Wu GC (2013). Meridian–viscera correlationship. Current research in acupuncture.

[40] Jenkinson M, Beckmann CF, Behrens TE, Woolrich MW, Smith SM (2012). FSL. Neuroimage.

[41] Zeng F, Lan L, Tang Y, Liu M, Liu X, Song W (2015). Cerebral responses to puncturing at different acupoints for treating meal-related functional dyspepsia. Neurogastroenterol Motil..

[42] Qiu K, Jing M, Sun R, Yang J, Liu X, He Z (2016). The status of the quality control in acupuncture-neuroimaging studies. Evid Based Complement Alternat Med.

[43] Nobis L, Manohar SG, Smith SM, Alfaro-Almagro F, Jenkinson M, Mackay CE (2019). Hippocampal volume across age: nomograms derived from over 19,700 people in UK Biobank. Neuroimage Clin..

[44] Zeng F, Sun R, He Z, Chen Y, Lei D, Yin T (2019). Altered functional connectivity of the amygdala and sex differences in functional dyspepsia. Clin Transl Gastroenterol.

[45] Hatta T (2007). Handedness and the brain: a review of brain-imaging techniques. Magn Reson Med Sci.

[46] Lichenstein SD, Verstynen T, Forbes EE (2016). Adolescent brain development and depression: a case for the importance of connectivity of the anterior cingulate cortex. Neurosci Biobehav Rev.

[47] Havsteen I, Ohlhues A, Madsen KH, Nybing JD, Christensen H, Christensen A (2017). Are movement artifacts in magnetic resonance imaging a real problem? A narrative review. Front Neurol.

[48] Cohen HA, Blau H, Hoshen M, Batat E, Balicer RD (2014). Seasonality of asthma: a retrospective population study. Pediatrics..

[49] Sharma VD, Sengupta S, Chitnis S, Amara AW (2018). Deep brain stimulation and sleep–wake disturbances in Parkinson disease: a review. Front Neurol.

